# Editorial: Ear-centered sensing: from sensing principles to research and clinical devices, volume II

**DOI:** 10.3389/fnins.2023.1327801

**Published:** 2023-11-17

**Authors:** Jérémie Voix, Preben Kidmose, Martin Georg Bleichner

**Affiliations:** ^1^Université du Québec (ÉTS), Montreal, QC, Canada; ^2^Department of Electrical and Computer Engineering, Aarhus University, Aarhus, Denmark; ^3^Department of Psychology, University of Oldenburg, Oldenburg, Lower Saxony, Germany

**Keywords:** EEG, brain activity, in-ear, brain-computer interface (BCI), wearable and mobile computing

Welcome to the second volume of the “*Ear-centered sensing: from sensing principles to research and clinical devices*” Research Topic. In this Research Topic of nine articles, we delve deeper into the realm of ear-centered sensing, exploring its multifaceted applications and shedding light on the immense potential this field holds. Our aim, as Topic Editors, is to provide you with a ome of latest research and developments within this emerging field.

## Ear-centered sensing: “the ear beyond hearing”

Ear-centered sensing, or “the ear beyond hearing” as coined by one of the Topic Editors (Voix, 2017), represents a groundbreaking approach to monitoring physiological signals, offering an unobtrusive and convenient means of data acquisition in everyday life. Physiological parameters such as heart rate, respiratory rate, eye blink and motion signals, skin conductance, as well as electrical activity from muscles and the brain, can all be captured from the ear. This unique positioning not only allows for discreet monitoring but also supports extended data recording, fostering a deeper understanding of psycho-physiological processes.

Ear-centered sensing is poised to play a pivotal role in scientific, diagnostic, and therapeutic endeavors, with a particular emphasis on mobile health applications. The challenges we face in this domain are intriguing, as we seek to bridge the gap between unconventional monitoring locations and established recording sites. Questions regarding signal fidelity, sensitivity, and real-time artifact discrimination remain at the forefront of our exploration.

## Advancements in sensor technology

Dedicated sensor and amplifier technology are paramount for successful long-term usage of ear-centered sensing devices. Authors that contributed their papers in this Research Topic have made significant strides in this area, striving for unobtrusiveness in every aspect. From biocompatible materials that adapt to individual anatomy to lightweight, inconspicuous instrumentation, their innovations promise to enable seamless data acquisition without restricting users' daily activities.

## The path forward: contributions to ear-centered sensing

In this volume II of our Research Topic, we present a diverse range of research articles that collectively advance the field of ear-centered sensing. We have compiled a brief overview of each article to pique your interest:

1. “*Ear-EEG measures of auditory attention to continuous speech*”: This study explores the potential of ear-centered EEG to monitor auditory attention during continuous speech, offering insights into assistive devices for complex auditory environments (Holtze et al.).

2. “*Evaluation of real-time endogenous brain-computer interface developed using ear-electroencephalography*”: Investigating the feasibility of real-time endogenous ear-EEG-based brain-computer interfaces, this article explores the potential of ear-EEG in online environments (Choi et al.).

3. “*In-ear electro-oculography for attended speaker estimation*”: Utilizing in-ear electro-oculography, this research focuses on improving comprehension in hearing-impaired individuals during conversations, with implications for hearing assistive devices (Skoglund et al.).

4. “*Pre-gelled electrode grid for self-applied EEG sleep monitoring at home*”: Addressing the need for convenient home sleep monitoring, this article presents a self-applicable EEG sensor array for accurate sleep evaluation (da Silva Souto et al.).

5. “*Assessing focus through ear-EEG*”: Investigating the potential of ear-EEG to determine levels of attention and focus, this study explores the integration of ear-EEG into wearable devices for monitoring mental load (Crétot-Richert et al.).

6. “*Synchronization of ear-EEG and audio streams in a portable research hearing device*”: This article assesses the alignment of audio and EEG data in the context of hearing aid algorithms, offering insights into future closed-loop EEG and audio applications (Dasenbrock et al.).

7. “*Sound localization in children with unilateral microtia and atresia*”: Exploring sound localization in children with hearing conditions, this study highlights the benefits of non-surgical bone conduction devices (Liu et al.).

8. “*At-home sleep monitoring using generic ear-EEG*”: Introducing a generic ear-EEG device for at-home sleep monitoring, this research emphasizes the potential for widespread sleep stage monitoring (Tabar et al.).

9. “*Ear-EEG sensitivity modeling for neural sources and ocular artifacts*”: This study establishes the sensitivity of ear-EEG to neural sources and ocular artifacts, supporting its integration into EEG paradigms (Yarici et al.).

As you delve into these nine articles, we hope you gain a deeper appreciation for the transformative potential of ear-centered sensing across various domains, from healthcare to cognitive science. By bringing together experts from different disciplines, we aim to foster collaboration and innovation in this every-day growing field.

## Author contributions

JV: Writing—original draft, Writing—review & editing. PK: Writing—review & editing. MB: Writing—review & editing.
